# An Iterative Shifting Disaggregation Algorithm for Multi-Source, Irregularly Sampled, and Overlapped Time Series

**DOI:** 10.3390/s25030895

**Published:** 2025-02-01

**Authors:** Colin O. Quinn, Ronald H. Brown, George F. Corliss, Richard J. Povinelli

**Affiliations:** 1 Department of Computer Science, Marquette University, 1313 W. Wisconsin Avenue, Milwaukee, WI 53233, USA; colin.o.quinn@marquette.edu; 2 Marquette Energy Analytics LLC, 313 North Plankinton Avenue, Suite 206, Milwaukee, WI 53203, USA; ron.brown@marquetteenergyanalytics.com; 3 Department of Electrical and Computer Engineering, Marquette University, 1515 W. Wisconsin Avenue, Milwaukee, WI 53233, USA; george.corliss@marquette.edu

**Keywords:** time series disaggregation, gas consumption disaggregation, nonuniform sampling, constrained redistribution, time series forecasting, multi-source, load shifting

## Abstract

Accurate time series forecasting often requires higher temporal resolution than that provided by available data, such as when daily forecasts are needed from monthly data. Existing temporal disaggregation techniques, which typically handle only single, uniformly sampled time series, have limited applicability in real-world, multi-source scenarios. This paper introduces the Iterative Shifting Disaggregation (ISD) algorithm, designed to process and disaggregate time series derived from sensor-sourced low-frequency measurements, transforming multiple, nonuniformly sampled sensor data streams into a single, coherent high-frequency signal. ISD operates in an iterative, two-phase process: a prediction phase that uses multiple linear regression to generate high-frequency series from low-frequency data and correlated variables, followed by an update phase that redistributes low-frequency observations across high-frequency periods. This process repeats, refining estimates with each iteration cycle. The ISD algorithm’s key contribution is its ability to disaggregate multiple, nonuniformly spaced time series with overlapping intervals into a single daily representation. In two case studies using natural gas data, ISD successfully disaggregates billing cycle and grouped residential customer data into daily time series, achieving a 1.4–4.3% WMAPE improvement for billing cycle data and a 4.6–10.4% improvement for residential data over existing methods.

## 1. Introduction to Temporal Disaggregation

A common challenge in forecasting is that the required forecast granularity often does not match the granularity of the observed data. For example, a daily forecast may be needed, but only historical monthly data is available. To address this, low-frequency time series data can be temporally disaggregated into higher frequency series. Current temporal disaggregation methods, however, typically transform only a single, uniformly sampled time series [1]. These approaches do not accommodate the disaggregation of multiple time series with nonuniform sampling.

This paper introduces the Iterative Shifting Disaggregation (ISD) algorithm, which processes natural gas flow measurements across multiple sensor-sourced time series and transforms the signal readings from low sampling rates to a high sampling rate. This transformation ensures coherence between the low and high sampling rates and incorporates the expected variability at the high sampling rate. Unlike traditional methods, the ISD algorithm can disaggregate multiple nonuniformly sampled time series into a single high-frequency signal while maintaining coherence across aggregation levels. Specifically, the disaggregated time series can be re-aggregated to match the original low-frequency data, preserving consistency.

In this article, we evaluate the effectiveness of the ISD algorithm in two case studies using natural gas data. The first study focuses on disaggregating low-frequency billing cycle time series from approximately monthly to daily frequency. The second case study focuses on disaggregating the time series of 100 residential customers’ monthly bills to obtain a single daily time series representing the aggregated consumption of all residents (i.e., neighborhood consumption). The primary goal of each evaluation is to transform disparate billing cycle time series into consistent daily time series, enabling more precise analysis and forecasting. The datasets used in these case studies reflect industry-standard disaggregation applications that can be generalized to utility distribution systems. We provide detailed structural descriptions of the data and methodology to ensure reproducibility using publicly available sources.

The ISD algorithm is an iterative two-phase process. The prediction phase uses multiple linear regression to generate high-frequency time series using the observable low-frequency time series and independent correlated high-frequency signals. The shifting phase, in a piecewise linear manner, redistributes low-frequency observations among high-frequency periods. This two-phase process continues iteratively, with each subsequent prediction phase modeling the updated estimates produced by the shifting phase.

Although ISD is presented in the context of natural gas consumption disaggregation, its methodology is not inherently restricted to this domain. The algorithm is formulated as a general-purpose disaggregation framework that can be adapted to other applications involving irregularly spaced time series, such as electricity demand forecasting, water usage analysis, or even sensor-based traffic flow modeling. However, its effectiveness in non-energy-related applications, such as image compression, would require additional domain-specific considerations.

The paper is structured as follows: First, the problem statement of this work is defined, and a survey of existing work in the field of temporal disaggregation is provided in Section 2. Section 3 describes the ISD algorithm. Section 4 compares the ISD algorithm to state-of-the-art temporal disaggregation methods. Finally, the conclusion summarizes the paper and suggests future improvements to the ISD algorithm.

## 2. Problem Statement and Prior Work

The temporal disaggregation problem arises when a low-frequency time series does not meet the granularity required for accurate forecasting or analysis. Section 2.1 describes the temporal disaggregation problem, while Section 2.2 reviews the literature pertinent to the Iterative Shifting Disaggregation (ISD) algorithm.

### 2.1. Temporal Disaggregation Problem

This section first presents the single-series disaggregation, and then the more general problem with multiple time series. Let Y be a low-frequency problem time series that is nonuniformly sampled,

(1) $$Y=\left\{Y_{i},\;i=1,\;\ldots ,\;m\right\},$$

and let y be the corresponding higher frequency time series that is uniformly sampled.

(2) $$y=\left\{y_{j},\;j=1,\;\ldots ,\;n\right\}.$$

**Figure 1:**
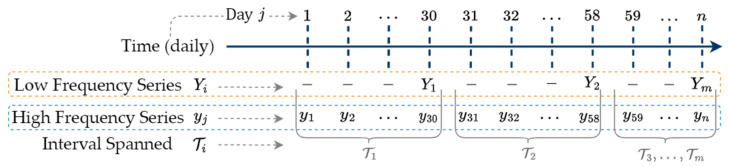
Low-frequency Y and high-frequency y time series.

Figure 1 illustrates an example temporal relationship between these two series, where $T_{i}$ is the interval that $Y_{i}$ spans.

Figure 1 shows time interval $T_{1}$ containing the first low-frequency observation $Y_{1}$ and the first thirty high-frequency observations $\left[y_{1},\;\ldots ,\;y_{30}\right]$. Similarly, time interval $T_{2}$ contains low-frequency observation $Y_{2}$ and high-frequency observations $\left[y_{31},\;\ldots ,\;y_{58}\right]$.

Let $T=[T_{1},\;T_{2},\;\ldots ,\;T_{m}]$ represent all m intervals of Y. Let A be an aggregation operator such that

(3) $$Y=A\left(y,T\right),\;\mathrm{where}\;\;Y_{i}=\sum_{j\in T_{i}}y_{j}.$$

Aggregation operator A sums the high-frequency time series y for each interval $T_{i}$ to yield low-frequency time series $Y=\{\;Y_{i}\}$. The disaggregation operator, $A^{-1}$, takes a low-frequency series and creates a corresponding high-frequency time series.

(4) $$y=A^{-1}\left(Y,T\right).$$

The ISD algorithm processes nonuniformly sampled data with irregular intervals ranging from 4 to 40 days. It also handles gaps in data by initializing missing values with zeros during the preparation step. This ensures alignment of time series for subsequent disaggregation and shifting phases. An example disaggregation would take a monthly time series (e.g., gas meter readings) and create the corresponding ~30 daily consumption estimates. The disaggregation operator $A^{-1}$ is not uniquely determined, so we rewrite (4) as

(5) $$\hat{y}=A^{-1}\left(Y,T\right)+\epsilon .$$

To address the under-constrained nature of $A^{-1}$ [2,3], additional constraints are applied to provide a near-unique definition of $A^{-1}$ [4]. Distributive disaggregation techniques constrain $A^{-1}$ such that sum of ${\hat{y}}_{j\in T_{i}}$ equals $Y_{i\;}$ [4,5]. This constrained relationship is referred to as forecast coherence and ensures that the high-frequency time series $\hat{y}$ maintains the same properties of the low-frequency observable Y [6]. Let J be the sum of square errors between y and estimate $\hat{y}$. Then, the forecast reconciliation constraint is

(6) $$\mathrm{minimize}\;J(y,\hat{y}):\;e_{j}={\left(y_{j}-{\hat{y}}_{j}\right)}^{2}$$

$$\mathrm{with}\;\mathrm{respect}\;\mathrm{to}\;\hat{y}\in A^{-1}\left(Y,T\right)$$

(7) $$\mathrm{subject}\;\mathrm{to}\;\;Y_{i}=\sum_{j\in T_{i}}{\hat{y}}_{j},\;{\hat{y}}_{j}\geq 0.$$

The disaggregation problem estimates high-frequency $\hat{y}$ to minimize the objective function $J(y,\hat{y})$ subject to the temporal aggregation constraints in (7) for all m intervals.

The ISD algorithm mimics a constrained optimization problem by iteratively minimizing J over $A^{-1}$ subject to the linear constraint (7). While the disaggregation operators described in Section 2.2 maintain consistency between the sum of the high-frequency estimates and the low-frequency actuals, traditional operators do not recreate the variability within the high-frequency estimates. This is especially true when working with datasets that contain measurements with significant temporal variability. Maintaining the consistency between temporal hierarchies is a way of ensuring the total of $\hat{y}$ agrees with Y and limits the solution space of $A^{-1}(Y)$. However, this constraint can limit the magnitude of $\hat{y}$, even though accurately estimating high-variance intervals is critical in many applications.

### 2.2. Review of Relevant Literature

Given time series Y, we want to estimate y, a higher frequency time series, i.e., forecast at a more granular level. The most accurate forecasts are achieved when the measurement frequency aligns with the forecast frequency demanded [7,8,9]. Temporal disaggregation generates additional, high-frequency historical data to better estimate parameters, reintroduces variability into a series that might have been smoothed through aggregation, and enables analysis at a previously unavailable resolution [5,10]. There are two general approaches to distributive disaggregation: using pure time series dynamic models (less common) and relying on independent correlated series at the desired frequency to obtain $\hat{y}$ (more common) [11]. This section begins with single time series dynamic models.

Pure time series dynamic disaggregation algorithms do not use exogenous information in evaluating A. These methods estimate $\hat{y}$ by assigning proportions of low-frequency measurements in Y to the underlying $\hat{y}$ so that the sum of squares error (6) is minimized. Chan published a simple, yet effective, approach known as naïve disaggregation [12]. Chan’s method disaggregates a yearly time series into quarterly values by using historical proportions to split annual values equally among quarters. This technique can be generalized to disaggregate any low-frequency interval to a higher frequency. However, Chan notes that this approach assumes no variation across the data and fails to capture the variability of the quarterly series. As a result, naïve disaggregation is often treated as a baseline method in the disaggregation literature and is commonly applied for single-series cases.

Wei and Stram address the challenge of recreating the variability absent in the naïve approach [10]. They develop an aggregate ARIMA model that leverages high-frequency data [13]. Using the autocovariance structure of this model, they estimate the unknown autocovariance structure of the unobserved disaggregated series. Once acquired, this structure reintroduces variability to the unobserved series. Wei and Stram show that the autocovariance structure of a model at high frequency can be derived uniquely from an aggregated model if the order of aggregation (number of summed intervals constituting the aggregated series) evenly divides the total number of observations in the aggregated series. Wei and Stram conclude that no disaggregation method can be optimal unless the intrinsic characteristics of a time series’ correlation are incorporated into the method [10]. Although their work examines autocorrelation within a single time series, many studies include correlated exogenous series in disaggregation.

Chow and Lin’s Generalized Least Squares (GLS) disaggregation method uses exogenous variables [14]. When auxiliary disaggregation information exists, it is preferable to employ a disaggregation procedure that combines all available (aggregated and disaggregated) information rather than using only aggregated data [15]. Similar to Chan [12], Chow and Lin disaggregate a series at yearly frequency to a series at quarterly frequency, which is common in econometrics [4]. However, rather than using historical proportions or lagged versions of the original series, they estimate GLS coefficients with correlated quarterly series, which generates variation in the disaggregated time series. To avoid linear bias, their method constrains the disaggregated data so it sums to the aggregated observations. Using multiple related high-frequency series has become a popular disaggregation approach [4].

Moauro introduces a state-space disaggregation method that uses seemingly unrelated structural time series (SUTSE) to estimate a higher frequency time series [5]. Moauro considers the SUTSE model strategy a more appropriate choice for their disaggregation work than the univariate regression-based approaches because of the SUTSE model’s flexibility, allowing for any kind of temporal disaggregation (e.g., yearly to monthly, monthly to daily, or daily to hourly) on raw or seasonally adjusted data. The robust SUTSE algorithm works well with the constraint that all intra-period data must sum to the original overlaying period. Additionally, Moauro’s approach differs from the traditional time series disaggregation approaches; disaggregation of the time series and the extraction of seasonal trends are done simultaneously. This simultaneous step makes Moauro’s SUTSE approach highly efficient and robust. However, in cases with high noise, other approaches may achieve better accuracy [16]. High noise can significantly impact the accuracy of time series disaggregation [17]. Previous studies have introduced methods for noise suppression that enhance model robustness and convergence in dynamic forecasting [18,19]. The ISD disaggregation algorithm introduced in this manuscript combines the ideas of pure time series dynamic models and methods that rely on independent correlated series to obtain $\hat{y}$.

The Time Series Reconstruction (TSR) algorithm [2] uses multiple related high-frequency time series in its disaggregation and was developed for the same purpose as our ISD method, i.e., for disaggregating natural gas consumption time series. TSR is a regression-based approach utilizing Generalized Least Squares as outlined by Chow and Lin [14]. TSR’s objective is to accurately disaggregate natural gas billing cycle data, especially during extreme cold events. Unlike other methods, TSR does not impose the forecast coherence constraint as defined in (7). This flexibility allows energy utilities to prioritize accurate forecasts during peak demand on cold days. TSR produces competitive results and inspired the development of the ISD algorithm presented here.

## 3. Iterative Shifting Disaggregation Algorithm

This section presents the Iterative Shifting Disaggregation (ISD) algorithm. The ISD algorithm disaggregates multiple data series structured at nonuniform, low levels of aggregation while maintaining the reconciliation constraint introduced in Section 2.1. ISD iteratively generates consumption load profiles based on the relationship between observable low-frequency series and high-frequency, independent correlated variables. The resulting high-frequency series are a product of “shifting”, or redistributing, the low-frequency observations among the high-frequency intervals. This process adjusts the initial naïve disaggregation using error estimates derived from correlated variables, ensuring that the high-frequency series captures day-to-day variability more accurately. ISD is presented in three steps, summarized in Algorithm 1.
**Algorithm 1:** Iterative Shifting Disaggregation**Inputs:****Multiple low-frequency**, inconsistently spaced, time series for disaggregation $x_{1},\;x_{2},\;\ldots ,\;x_{P}$—*P* independent correlated variables measured at the target frequency of y 
nLRModels—Hyperparameter (integer) used to control the number of linear regression models trained in the disaggregation process 
nDisaggCycles—Hyperparameter (integer) used to control the number of times load is shifted among underlying intervals α—Parameter (float) used to control how error is redistributed in disaggregation process**Output:**$\hat{y}$—An estimate of the high-frequency, consistently spaced, disaggregated time series 
1 2 3Naïvely disaggregate multiple input series and aggregate into daily time series $\hat{y}$ Form design n×P matrix $X=\left[x_{1},x_{2},\;\ldots ,\;x_{P}\right]$ of transformed independent correlated variables
4**for** (nLRModels) **do**
5 6 7 Prediction Phase: Model daily $\hat{y}$ using exogenous variables X produce ${\hat{\beta }}_{p}$ Evaluate $X\cdot {\hat{\beta }}_{p}$ to produce $\tilde{y}$

8 9
  **for** (nDisaggCycles) **do //** Each iteration “shifts” interval load among underlying days
10

 **for** (mIntervals) **do**
11 12 13 14 15


  Update Phase: Remove kth interval from aggregate $\hat{y}$, calculate error $\epsilon =\tilde{y}-\hat{y}$
Distribute error α·ϵ error into kth interval of $\hat{y}$ // Constraints must be considered, original interval still removed Add kth interval back aggregate $\hat{y}$

16


**end //** mIntervals
17

**end //** nDisaggCycles
18
**end //** nLRModels

The iterative shifting process consists of multiple nested loops, each serving a specific function in refining the disaggregated time series.

- The outer loop iterates over multiple regression models trained at different stages of disaggregation, allowing progressive refinement.
- The middle loop performs several disaggregation cycles, incrementally redistributing the error signal across high-frequency periods.
- The inner loop iterates over all input series (e.g., *A*, *B*, *C*, …), ensuring that each interval’s contribution is updated independently before restoring the full series.

The ISD algorithm accepts a list of one or more low-frequency time series measuring the same dependent variable. A design matrix is formed for the low-frequency time series. The P high-frequency time series $x_{p}$ are the exogenous, correlated time series. The term ‘design matrix’ follows standard usage in statistical learning and regression modeling. It refers to a structured representation of explanatory variables, where each row corresponds to an observation (e.g., a day in the disaggregated series), and each column represents a predictor variable. The predictors in *X* include exogenous variables such as Heating Degree Days (HDD), Cooling Degree Days (CDD), and Heating Degree Days with Wind (HDDW), which influence natural gas consumption patterns. By organizing the data in this matrix form, the regression framework can efficiently estimate coefficients and generate high-frequency predictions.

ISD depends on the correlations between the exogenous variables and the dependent high-frequency time series. Control parameters nLRModels, nDisaggCycles, and α are user-defined inputs used in the prediction and update phases. nLRModels specifies the number of gas consumption profiles generated in the prediction phase, while nDissagCycles controls how many permutations occur over the overlapping intervals of the multiple low-frequency time series per LRModel. Parameter α is used to determine how much weight should be given to the current-state estimate when the shifting occurs in the update phase. Lastly, control-loop parameter mIntervals depends on the number of intervals in the multiple low-frequency time series and is calculated in Step 1. These parameters were selected based on empirical validation and best practices in time series disaggregation. Their specific values are tuned for each dataset based on historical error analysis and model performance assessments.

To further enhance reproducibility, Appendix A provides a structured breakdown of the ISD algorithm’s workflow. This pseudocode outlines the iterative prediction and adjustment process, enabling practical implementation using alternative datasets with similar characteristics. For the remainder of the ISD description, and without loss of generality, we assume the desired high frequency is daily, the low frequency consists of nonuniformly sampled intervals of multiple days, and there are three low-frequency time series: A, B, and C. For example, suppose energy is supplied to a set of consumers through three sources. Which sources supply energy on a given day is unknown, but the total energy supplied over intervals of days is measured. These intervals are not synchronized across their sensor-derived sources.

### 3.1. Step 1—Preparation of Time Series Inputs Using Low-Frequency Sampling

We extend the notation introduced in Section 2 to accommodate the multiple time series. For illustration, we will use time series A, B, and C. Each input time series (i.e., A, B, and C) represents independent sources with different temporal resolutions, requiring alignment before further disaggregation. These sources may originate from separate operational zones, customer groups, or metering systems, contributing distinct load profiles. The ISD framework first aligns these datasets by mapping their irregularly spaced observations to a common daily time axis before applying disaggregation. The objective is to estimate coherent $\hat{y}$, which is the daily sum of the multiple time series. Let A be a time series with index i and $N_{A}$ observations

(8) $$A=\left\{A_{i},\;i=1,\;\ldots ,\;N_{A}\right\}.$$

**Figure 2:**
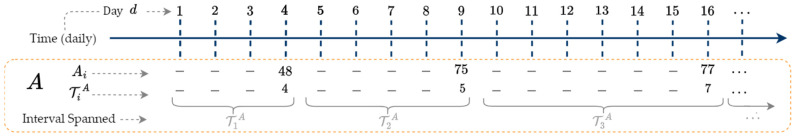
Time series $A_{i}$, interval durations $T_{i}^{A}$, and daily index d.

The time steps of A may span long, nonuniform durations, anywhere between 4–40 days. Let $T_{i}^{A}$, also indexed by i, represent the number of days each $A_{i}$ observation spans (similar to Figure 1’s $T_{i}$, however now a single integer of days). Let d be a daily index used to denote the days on which each observation $A_{i}$ occurs. Index d later serves to synchronize multiple time series to a common time step. Figure 2 illustrates $A_{i}$, d, and $T_{i}^{A}$.

Figure 2 shows the first observation $A_{1}$ = 48 occurs on the fourth day (d = 4) and is measured over a four-day interval ($T_{1}^{A}\;$ = 4). Thus, $A_{1}$ is defined on the time interval [1,4] as illustrated by the gray bracket. The general time interval for each $A_{i}$ is $\left[d-T_{i}^{A}+1,d\right]$, where d is the day number over which $A_{i}$ is aggregated. The second gas consumption observation $A_{2}$ = 75 spans five days ($T_{2}^{A}=5)$ and is on the time interval [5,9].

The first step of the ISD algorithm naïvely disaggregates A into a daily series [12]. To enhance reproducibility, this section provides details on how input time series are structured and preprocessed before applying ISD. Each time series consists of irregularly spaced interval measurements, which are first aligned to a common time domain using the naïve disaggregation method. The transformation follows Equation (9), ensuring that each interval’s total consumption remains preserved. Naïve disaggregation creates a daily representation of an interval series, while maintaining the consistency constraints that the underlying days of the high-frequency disaggregated series sum to the interval observation from which they originate. This is achieved by dividing each $A_{i}$ by $T_{i}^{A}$ to get an average value for all days within the ith interval. Missing data in low-frequency time series, such as a missing monthly bill, are estimated using a low-frequency regression model based on similar intervals or correlated variables to avoid bias introduced by assuming zero values. Once estimated, the interval is divided evenly across its days, maintaining alignment during iterative updates. For example, if a missing monthly value is estimated as 100 and spans 5 days, 20 units are allocated per day. We augment $A_{i}$ with the day d on which it occurred, yielding $A_{i,d}$. The naïvely disaggregated series, $\bar{a}$ indexed by i,j is calculated as

(9) $${\bar{a}}_{i,j}=\frac{A_{i,d}}{T_{i}^{A}},\;where\;j=d-T_{i}^{A}+1,\;\ldots ,\;d\;and\;i=1,\;\ldots N_{A}.$$

**Figure 3:**
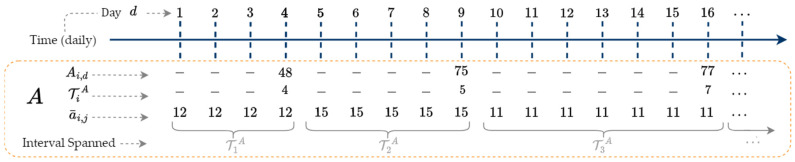
Time series A naïvely disaggregated into daily series $\bar{a}$.

Figure 3 shows an example series $\bar{a}$ created from the A shown in Figure 2.

Index j in (9) is used to distribute each observation $A_{i,d}$ equally among the $T_{i}^{A}$ days spanned by interval i. The index i is used to keep track of the interval of ${\bar{A}}_{i,d}$. For example, the first interval observation $A_{1,4}$ creates disaggregated ${\bar{a}}_{1,j}=\frac{48}{4}=12,\;for\;j\in [1,\;2,\;3,\;4]$. The next interval observation $A_{2,9}$ creates disaggregated ${\bar{a}}_{2,j}=\frac{75}{5}=15,\;for\;j\in [5,\;6,\;7,\;8$, 9].

We now consider the case when multiple low-frequency time series are used as inputs to ISD. Given an additional time series B, indexed by k with $N_{B}$ observations, and C, indexed by l with $N_{C}$ observations, then

(10) $$B=\left\{B_{k},\;k=1,\;\ldots ,\;N_{B}\right\},$$

(11) $$C=\left\{C_{l},\;l=1,\;\ldots ,\;N_{C}\right\}.$$

Repeating the process of creating $\bar{a}$, we create $\bar{b}$ and $\bar{c}$.

**Figure 4:**
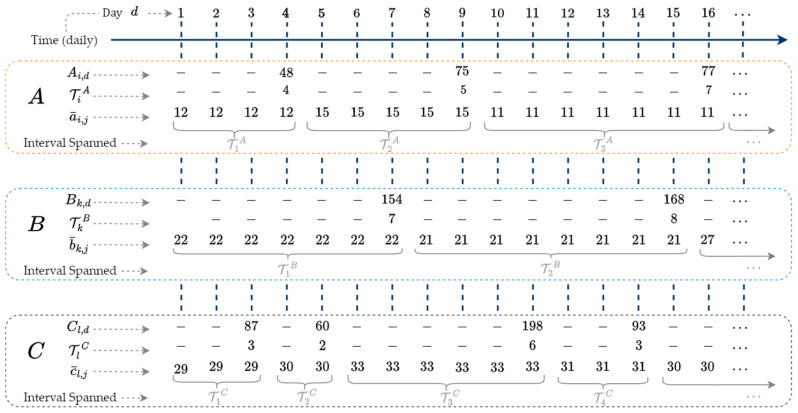
Three input time series A, B, and C with overlapping time intervals.

A, B, and C are defined on the same time domain and may have overlapping intervals. Figure 4 illustrates the overlapping intervals of each series and presents example series $\bar{b}$ and $\bar{c}$. In practice, the interval lengths of B and C span longer durations than pictured.

The example series in Figure 4 have nonuniform time steps and overlapping intervals. We replace all missing/unaccounted for observations throughout $\bar{a}$, $\bar{b,}$ and $\bar{c}$ with a value of zero, forcing each series to have an equal number of days. We assume each time series starts and ends on the same day.

We aggregate each day’s average within $\bar{a}$, $\bar{b}$, and $\bar{c}$ to create an initial estimate of the daily time series $\hat{y}$ indexed by days d. Aggregate $\hat{y}$ as

(12) $$\hat{y}=\bar{a}+\bar{b}+\bar{c}.$$

**Figure 5:**
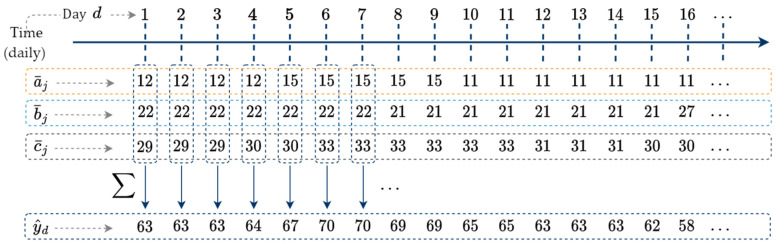
Each day d is aggregated into the ${\hat{y}}_{d}$ daily time series.

The aggregation in (12) is shown in Figure 5.

Figure 5 shows how each day d is aggregated into the ${\hat{y}}_{d}$ daily time series. For example, on day one (d=1), ${\bar{a}}_{1}=12$, ${\bar{b}}_{1}=22$, and ${\bar{c}}_{1}=29$ are aggregated to form ${\hat{y}}_{1}=63$. On $d=7,\;{\bar{a}}_{7}=15$, ${\bar{b}}_{7}=22$, and ${\bar{c}}_{7}=33$ are aggregated to form ${\hat{y}}_{7}$ = 70. Naïve disaggregation maintains coherence in the construction of $\hat{y}$, meaning that the consistency constraint of $\bar{a}$, $\bar{b}$, and $\bar{c}$ is met in $\hat{y}$. We now consider the independent correlated variables used to generate gas consumption profile estimates in Step 2.

### 3.2. Step 2—Iteratively Modeling Daily Time Series (High-Frequency Sampling)

The exogenous variables are sampled daily and on the same time domain as $\hat{y}$. ISD depends on a strong correlation between the exogenous variables and dependent variables. Let $N_{D}$ represent the total number of days within $\hat{y}$. The exogenous variables incorporated in ISD include:

- Heating Degree Days (HDD): A temperature-based metric that quantifies heating demand by measuring how much daily temperatures fall below a base threshold.
- Cooling Degree Days (CDD): A complementary metric that captures cooling demand based on how much daily temperatures exceed a base threshold.
- Wind-Adjusted Heating Degree Days (HDDW): An adjusted metric that accounts for wind speed effects on perceived temperature, improving heating load estimates.

These exogenous variables align with established forecasting models in natural gas research. While specific proprietary data sources were used in this study, alternative datasets such as NOAA’s publicly available temperature records can serve as suitable proxies for replicating the ISD methodology.

Given daily temperature series t and wind speed series w, both indexed by d with the same number of days ($N_{D})$ as $\hat{y}$

(13) $$t=\left\{t_{d},\;d=1,\;\ldots ,\;N_{D}\right\},$$

(14) $$w=\left\{w_{d},\;d=1,\;\ldots ,\;N_{D}\right\},$$

a nonlinear transformation is applied to these variables to obtain Heating Degree Days (HDD), Wind-Adjusted Heating Degree Days (HDDW), and Cooling Degree Days (CDD). To transform these weather time series, a reference temperature $T_{ref}$ typically between 55 and 65 °F is used, such that

(15) $$HDD_{d}=max(T_{ref}-t_{d},0),$$

(16) $$HDDW_{d}=\left\{\begin{array}{c} HDD_{ref}\frac{152+w_{d}}{160}\;w_{d}\leq 8\\ HDD_{ref}\frac{72+w_{d}}{80}\;w_{d}>8 \end{array}\right.$$

(17) $$CDD_{d}=\max\left(0,t_{d}-T_{ref}\right),$$

for all $N_{D}$ days. Transpose each resulting exogenous series from (15)–(17) using $T_{ref}$ = 65 and 55 °F to column vectors.

The selection of exogenous variables is guided by two key principles: relevance to the underlying consumption patterns and statistical robustness. Variables such as Heating Degree Days (HDD) and Cooling Degree Days (CDD) are chosen based on their established relationships with gas consumption in temperature-sensitive regions. Correlation analysis is conducted to confirm strong linear relationships between candidate variables and the target time series. Additionally, cross-validation techniques are applied to ensure that the inclusion of these variables improves predictive accuracy without introducing spurious correlations. For non-temperature-sensitive datasets, alternative variables such as socioeconomic indicators, day-of-week effects, or calendar-based seasonality may be employed, guided by domain-specific insights.

Form the design matrix *X* column wise using the P exogenous variables. For example, using P=4, exogenous weather variables HDD65, HDD55, HDDW65, and CDD65 [20] form design matrix X.

Design matrix X is an $N_{d}$ by P+1 matrix, where $N_{d}$ is the number of days in the daily aggregate series $\hat{y}$, and P is the number of independent correlated daily variables. Include a bias vector of 1’s in the first column of X to capture the natural gas consumption that is independent of temperature (base load) [21]. The exogenous degree-day variables shown in [22] are good indicators of future natural gas demand [9,23,24]. The generalized ISD algorithm accepts any number of exogenous variables.

The naïve disaggregation described in Step 1 fails to recreate the variability seen in an actual daily series. The remaining steps of the ISD algorithm iteratively adjust $\hat{y}$ to capture this variability. Shifting occurs in two phases: a prediction phase generates the a priori state estimate $\tilde{y}$ and an a posteriori update phase that shifts $\hat{y}$ based on the a priori state estimate. The prediction phase is the first iterative step of the ISD algorithm seen in line 2 in Algorithm 1. Let β be a vector of regression coefficients. In each iteration, a multiple linear regression model is fit using daily gas consumption estimates $\hat{y}$ and design matrix X to estimate $\hat{\beta }$. Estimate β using least squares, minimizing the mean square of residuals of all $N_{D}$ days

(18) $$\underset{\beta }{\min}\left\|\hat{y}-X\beta \right\|.$$

Use the estimated coefficients $\hat{\beta }$ to calculate daily load profile

(19) $$\tilde{y}=X\beta .$$

Series $\tilde{y}$ is this iteration’s a priori state estimate, and does not meet the coherency constraint [25].

Design matrix X remains constant across all iterations. Once the constrained shifting in $\hat{y}$ is carried out in Step 3, a new version of $\hat{y}$ is modeled with X in (19) to summarize the updated relationship between the daily gas consumption estimates and correlated variables in future iterations.

### 3.3. Step 3—Constrained Load Shifting

Step 3 iteratively adjusts daily series $\hat{y}$ towards estimated daily series $\tilde{y}$. The update in $\hat{y}$ maintains the temporal disaggregation reconciliation constraint that the disaggregated days ${\hat{y}}_{d}$ sum to the observed interval observation. In the case of multi-source data, ISD applies shifting corrections independently to each source before merging their refined estimates into the final disaggregated time series. This ensures that discrepancies in one source do not disproportionately affect another, maintaining the integrity of distinct load profiles while enforcing coherence in the overall disaggregation process. A, B, and C are the original interval observations. Recall that $\bar{a}$, $\bar{b}$, and $\bar{c}$ are initially naïvely disaggregated daily counterparts, respectively. In each iteration of this adjustment, the daily values are “shifted” within each interval to reflect the variability existing in correlated variables X.

**Figure 6:**
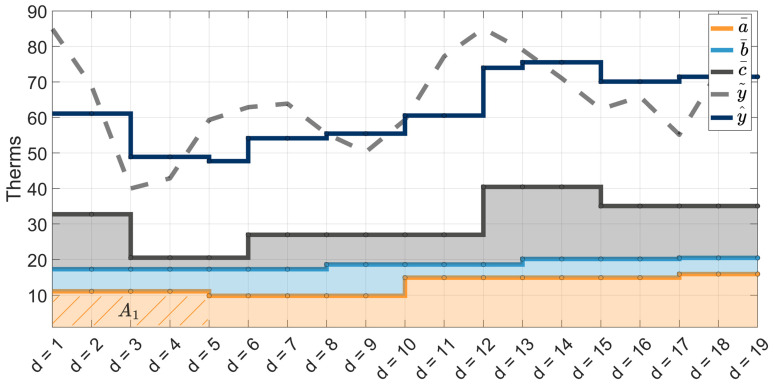
Plot of $\hat{y},$ formed from daily aggregation of $\bar{a}$, $\bar{b}$, and $\bar{c}$, compared to Step 3’s estimated load profile $\tilde{y}$ before any shifting occurs.

Daily time series $\hat{y}$ and $\tilde{y}$ are plotted in Figure 6 along with the values of naïvely disaggregated $\bar{a}$, $\bar{b}$ and $\bar{c}$ shown beneath to show all available daily information used in the ISD constrained shifting step.

The constrained shifting step of ISD iterates over all intervals of original input series A, B, and C. The following example of ISD’s constrained shifting is carried out using the first interval $A_{1}=48$, which spans four days over the time interval [1,2,3,4]. This information is directly reflected in the same four days of $\bar{a}$, where the sum of ${\sum_{d=1}^{4}\bar{a}}_{d}$ is constrained to equal $48=A_{1}$ (Figure 6—gold hash marks). The value of $A_{1}$ is a reconciliation constraint. The shifted values are then added into $\hat{y}$ at the end of each iteration.

Begin the shifting by removing the contribution of $A_{1}$ from $\hat{y}$ over the d days $A_{1}$ spans

(20) $${\hat{y}}_{d}={\hat{y}}_{d}-{\bar{a}}_{d}\;\mathrm{for}\;d=1,\;\ldots ,\;4.$$

The remaining values in ${\hat{y}}_{d}$ for days d ∈[1, …, 4] represent the overlapping interval contributions of $\bar{b}$ and $\bar{c}$. Calculate the error $\epsilon_{d}$ between $\tilde{y}$ and $\hat{y}$ over the same days

(21) $$\epsilon_{d}={\tilde{y}}_{d}-{\hat{y}}_{d}\;\mathrm{for}\;d=1,\;\ldots ,\;4.$$

Error $\epsilon_{d}$ is the difference between profiles of $\tilde{y}$ and $\hat{y}$ with the contribution of $A_{1}$ removed. It can be thought of as the estimated variability missing from naïvely produced interval ${\bar{a}}_{d}$. We constrain $\epsilon_{d}$ to be a nonnegative value by replacing negative errors with zero. The profile of ${\bar{a}}_{d}$ with error $\epsilon_{d}$ incorporates variability into new interval

(22) $$z_{d}=\frac{\epsilon_{d}*\sum_{j=1}^{4}{\bar{a}}_{j}}{\sum_{j=1}^{4}\epsilon_{j}},\;for\;d=1,\;\ldots ,\;4.$$

Temporary interval z can be thought of as $A_{1}$ shifted by the day-to-day variability extracted from the system as a whole. Some of the variability captured in temporary interval z may originate from components independent of A (e.g., overlapping intervals of B and C).

The exact day-to-day variability of $\bar{a}$ in relation to $\bar{b}$ and $\bar{c}$ is unknown, and as such, ISD updates $\bar{a}$ in small increments. Update $\bar{a}$ over the days spanned in $A_{1}$ as a weighted combination of $\bar{a}$’s original state and z. Let α be the weight used in this combination

(23) $${\bar{a}}_{d}=\left(1-\alpha \right)\left({\bar{a}}_{d}\right)+\left(\alpha \right)\left(z_{d}\right)\;for\;d=1,\;\ldots ,\;4.$$

**Figure 7:**
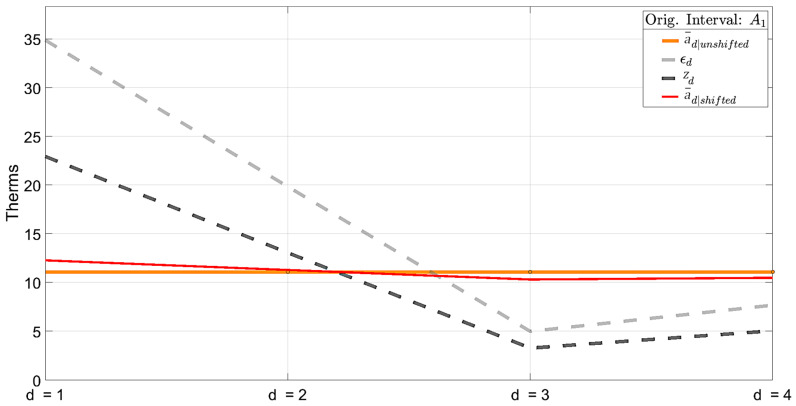
Newly updated interval ${\bar{a}}_{d|shifted}$, original ${\bar{a}}_{d|unshifted}$, constrained interval estimates $z_{d}$, and error $\epsilon_{d}$.

The newly updated interval within ${\bar{a}}_{d}$ is plotted in Figure 7 as ${\bar{a}}_{d|shifted}$. Also plotted are the values of ${\bar{a}}_{d}$ before any shifting occurs (${\bar{a}}_{d|unshifted}$), error $\epsilon_{d}$ (21), and interval estimate $z_{d}$ (22).

**Figure 8:**
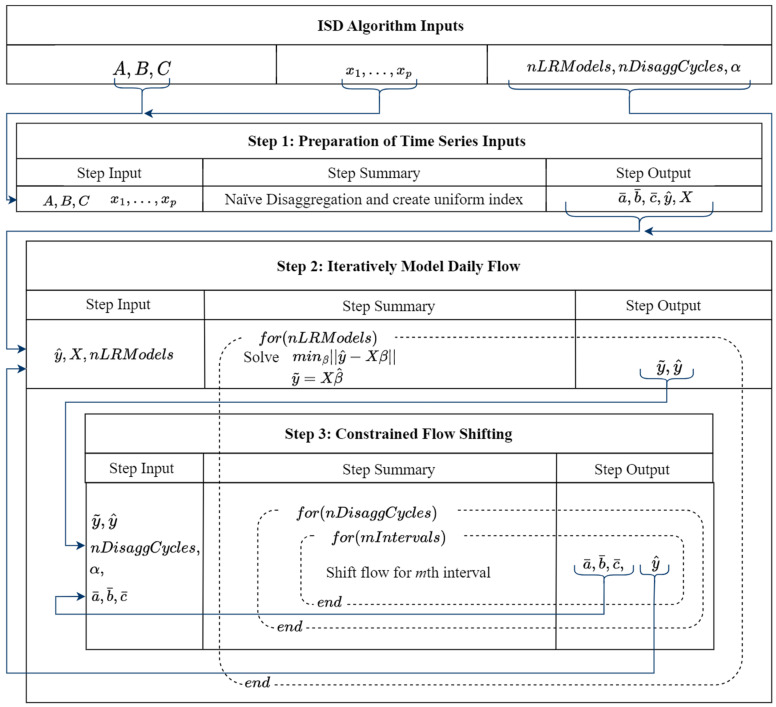
Three gas consumption load shifting steps carried out by ISD algorithm.

Step 3 carries out the shifting of a single interval example $A_{1}$ for a single disaggregation cycle. After all nDisaggCycles disaggregation cycles complete, a new relationship is modeled in the outermost for-loop of Figure 8.

The control-loop and gain variables nDisaggCycles, nLRModels, and α in Figure 8 are empirically determined and based on the number of interval series input to the ISD algorithm. If there are three or more input time series, nDisaggCycles=nLRModels=10, and α=0.05. If there are fewer than three inputs, more iterations are required. These variables may be adjusted for different applications.

**Figure 9:**
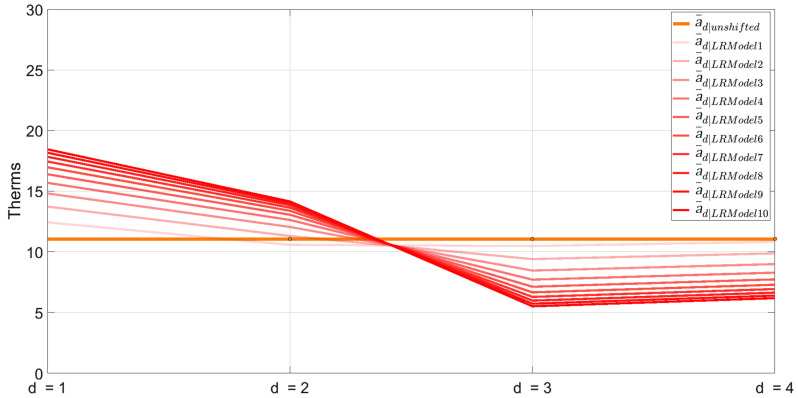
Evaluation of $A_{1}$ across all nLRModels. The orange line represents daily series ${\bar{a}}_{d|unshifted}$, while the gradient red line illustrates the progressive refinement of this interval after each regression model is trained.

Figure 9 shows how example interval $A_{1}$ shifts over each regression evaluation as the ISD algorithm determines the proportion of gas consumption to assign in each underlying day.

Daily series ${\bar{a}}_{d|unshifted}$ is the naïvely disaggregated version of $A_{1}$ before any shifting occurs. ${\bar{a}}_{d|LRModel1}$ is the first permutation made and the same as ${\bar{a}}_{d|shifted}$ in Figure 7. This shifting process is repeated for all remaining intervals in A, B, and C, then repeated for a total of nLRModel iterations.

## 4. Disaggregation Case Studies

Four temporal disaggregation methods are compared in the following case studies: Iterative Shifting Disaggregation algorithm (ISD—Section 3), naïve (NAV—Section 3.1), Time Series Reconstruction (TSR—[26]), and Generalized Least Squares (GLS—[10]). We present an analysis comparing each disaggregation technique with the others in the following section.

Two case studies are examined. The first study, which we refer to as the Operating Area case study, focuses on disaggregating low-frequency operating area billing cycle time series from an approximately monthly cycle frequency to a daily frequency. The Operating Area dataset consists of aggregated gas flow measurements from multiple distribution zones within a utility’s network. These data are sampled at irregular intervals based on billing cycles and operational reporting schedules. The dataset structure aligns with typical industry datasets used for forecasting and load analysis. Given the proprietary nature of the data, a detailed breakdown of its format and structure has been provided to ensure that researchers can apply the ISD methodology to alternative datasets with similar characteristics.

The second case study, referred to as Residential 100, focuses on disaggregating 100 residential customers series to daily time series. The Residential 100 dataset used in this study consists of anonymized, aggregated natural gas consumption data collected from 100 residential customers. The Residential 100 dataset reflects industry-standard customer billing structures and is representative of natural gas distribution data encountered by local distribution companies (LDCs).

**Figure 10:**
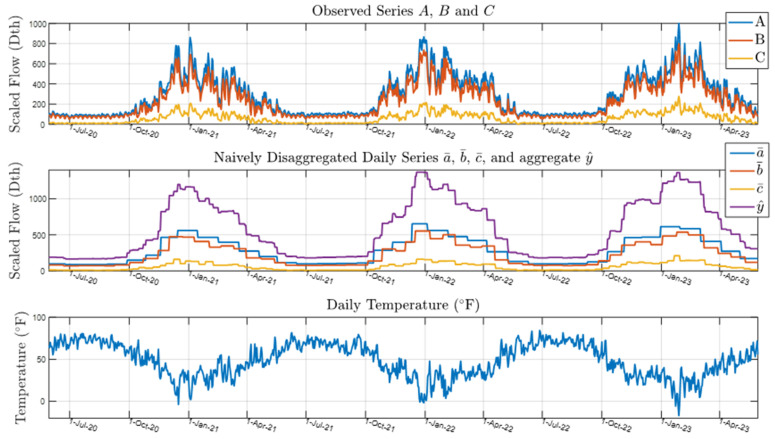
Plot of input time series A, B, and C over 2021–2023 heating seasons.

We used three low-frequency time series (A, B, C) with overlapping and varying interval lengths as inputs to each disaggregation algorithm. This study follows the description given in Section 3—Iterative Shifting Disaggregation Algorithm. The observed values of Operating Area times series A, B, and C from 2021–2024 are shown in the top plot of Figure 10. These series exhibit seasonal patterns, peaking during winter, indicating higher gas demand in colder months. The naïvely disaggregated time series $\bar{a},\;\bar{\;b}$, $\bar{c}$ and their aggregate $\hat{y}$ (result of Step 1) are shown in the middle plot. These disaggregated series also show seasonal fluctuations, mirroring the patterns seen in the observed data. The bottom plot shows daily temperature ($F^{\circ })$, revealing an annual cycle of higher summer temperatures and lower winter temperatures. The inverse relationship between temperature and gas demand is evident: gas consumption increases in winter as temperatures drop and decreases in summer when temperatures rise.

**Figure 11:**
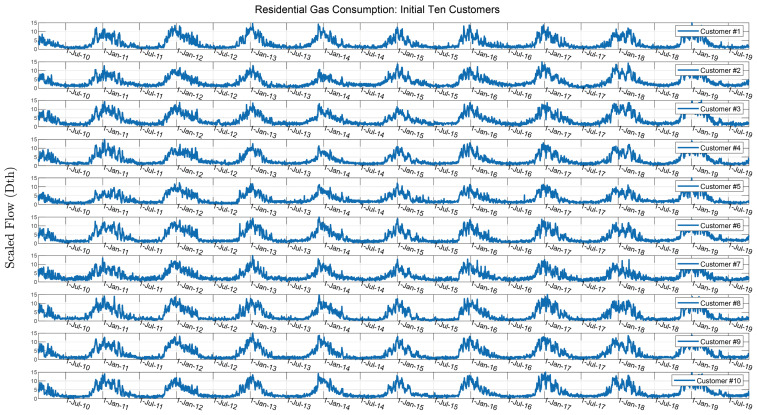
Plot of first ten of 100 customers consumption time series from Residential 100 dataset.

Figure 10 shows gas consumption from the three independently metered areas displaying periodic and cyclical characteristics. In the Residential 100 case study, we add a layer of granularity to the disaggregation problem by focusing on individual residential gas consumers. Similar patterns are observed in this dataset; however, instead of analyzing gas consumption across different geographical levels (e.g., neighborhoods like A, B, and C), we disaggregate the data for 100 individual consumers in a less temperature-sensitive area. The first ten customer gas consumption time series are plotted in Figure 11. The Residential 100 dataset consists of overlapping and incoherent customer time series.

We used a regression model with variables commonly used in gas demand forecasting, calculated similarly to those in [9,23,24] for incorporating exogenous variables. Based on these works, domain knowledge, and best practices from utilities’ forecasting in Vitullo et al. [2], eight correlated variables were used in model training: a linear trend, Heating Degree Days HDD65 and HDD55, Wind-Adjusted Heating Degree Day HDDW65, Cooling Degree Day CDD65, and four seasonal variables representing the first two harmonics of a Fourier series. Daily temperature and wind are transformed into $HDD^{*}$ and CDD variables using (15)–(17). The four seasonal variables are yearly periodic functions representing the first two harmonics of a Fourier series [22]. These exogenous variables were formatted into design matrix X as described in Section 3.1. The ISD algorithm can accept any number of exogenous variables, with the first iteration initializing these variables into design matrix X.

Three measures were used to summarize the performance of each algorithm: root-mean-square error (RMSE), mean absolute percentage error (MAPE), and weighed mean absolute percentage error (WMAPE). RMSE is a measure of the average residual. Let $y_{d}$ be the value of signal y at time *d*, ${\hat{y}}_{d}$ be the disaggregated quantity on day d, and $N_{d}$ be the total number of underlying days, then

(24) $$RMSE=\sqrt{\frac{\sum_{d=1}^{N_{D}}{\left({\hat{y}}_{d}-y_{d}\right)}^{2}}{N_{D}}}.$$

RMSE is measured in dekatherms. MAPE is the average absolute error. It is unitless and represents the percent of error.

(25) $$MAPE=\frac{1}{N_{D}}\sum_{d=1}^{N_{D}}\left|\frac{y_{d}-{\hat{y}}_{d}}{y_{d}}\right|\times 100.$$

Lastly, weighted mean absolute error (WMAPE) is commonly used to evaluate gas forecasting performance and weights the heating season more than the summers.

(26) $$WMAPE=\frac{\sum_{d=1}^{N_{D}}\left|y_{d}-{\hat{y}}_{d}\right|}{\sum_{d=1}^{N_{D}}\left|y_{d}\right|}\times 100.$$

The NAV, TSR, and GLS disaggregation methods do not share the ISD algorithm’s advantage of being able to simultaneously disaggregate multiple time series with overlapping intervals. Therefore, the low-frequency operating-area time series A, B, and C, as well as the 100 residential customer billing cycle time series, are disaggregated individually and aggregated post-process for all days d.

Table 1 presents the RMSE, MAPE, and WMAPE for each disaggregation algorithm applied to the Operating Area dataset.

**Figure 12:**
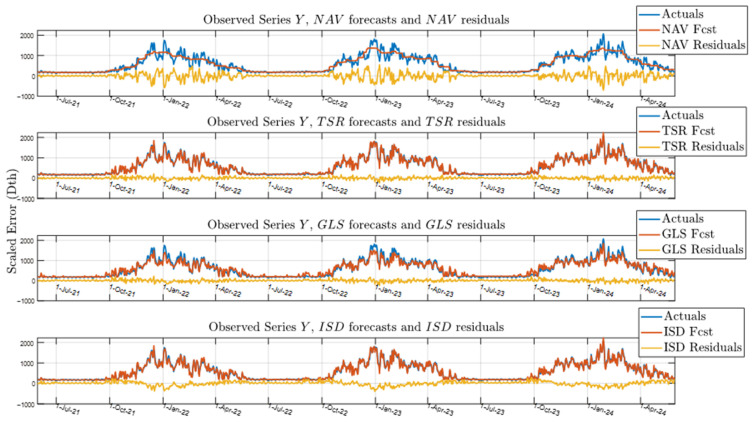
Residual comparison of actuals vs forecasts for NAV, TSR, GLS, and ISD disaggregation algorithms (Operating Area).

Table 1 shows that the ISD algorithm has the lowest MAPE, RMSE, and WMAPE, making it the most accurate disaggregation method when applied to the low-frequency Operating Area dataset. As expected, the NAV benchmark model is the least accurate. The TSR algorithm is the second most accurate, outperforming GLS, but still has higher error metrics than ISD. We examine the estimated daily series and residual errors for each disaggregation algorithm during the 2021–2023 heating seasons in Figure 12.

Figure 12 compares the different disaggregation methods (${\hat{y}}_{d}$—orange) with the actual observed values ($y_{d}$—blue). The residual difference between these series is plotted beneath (yellow) for each day d. The plot highlights how each method captures seasonal variability, showing higher values during peak periods and lower values during off-peak periods. Differences between the forecast methods and the actual values are more noticeable during periods of high variability, indicating differences in accuracy. The top NAV plot of Figure 12 illustrates this as the naïve disaggregation smooths out consumption across each heating season. Accurate forecasts are especially critical during high variability periods, such as winter [22]. The GLS and ISD plots of Figure 12 show better performance over these heating seasons, while the TSR algorithm shows the smallest residuals. This is examined further in Section 5.

The RMSE, MAPE, and WMAPE for each algorithm for the Residential 100 case study are presented in Table 2.

Table 2 shows that the ISD method outperforms the other disaggregation algorithms across all error metrics, achieving the lowest RMSE (6.61 Dth), MAPE (11.13%), and WMAPE (9.07%). This suggests that ISD’s coherent structure likely enhances its overall accuracy, especially in relative error terms. Despite its coherent structure, NAV shows the highest RMSE (20.32 Dth), indicating that coherence alone does not ensure minimal error in absolute terms. The high error values for NAV, with MAPE at 37.14% and WMAPE at 33.82%, are reasonable for a benchmarking naïve baseline, which serves as a reference point rather than an optimized solution. By contrast, TSR and GLS, though non-coherent, demonstrate moderate error rates, with TSR achieving lower WMAPE (13.3%) than GLS (19.43%), suggesting that TSR may better manage specific types of errors. Overall, these results underscore ISD as the most reliable disaggregation method.

**Figure 13:**
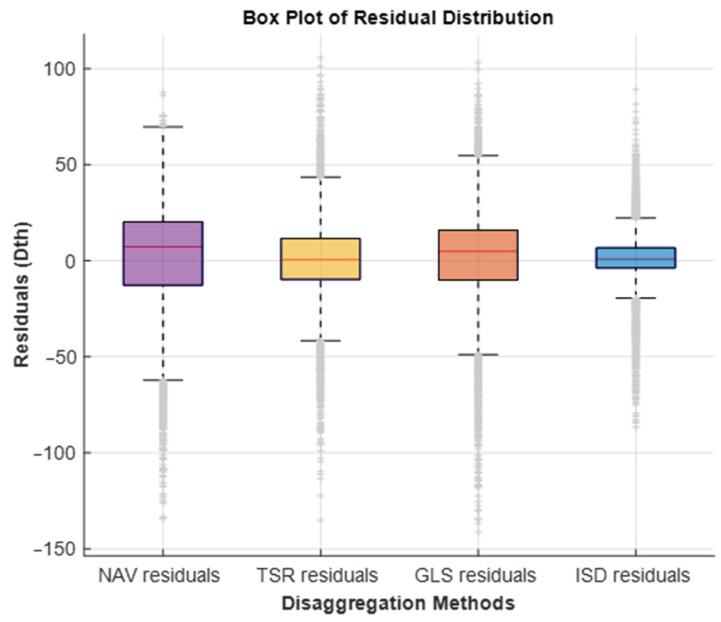
Residential 100 residuals analysis for NAV (purple), ISD (blue), TSR (yellow) and GLS (orange) disaggregation algorithms.

The data used in Residential 100 is considerably less temperature sensitive than the A, B, and C Operating Area dataset. We examine residential gas forecasting residual distribution for each method on the Residential 100 data in Figure 13.

Figure 13 reflects the results seen in Table 2, with the NAV persistence model having the largest spread of residual error in purple. The second largest interquartile range is shown in orange and is the GLS residual distribution. The TSR (yellow) and ISD (blue) have narrower residual spreads, with ISD algorithm performing best overall in the Residential 100 data disaggregation. Further conclusions are drawn between the temperature dependence between the A, B, and C Operating Area and Residential 100 datasets, and how they relate to each set of residuals in Section 5.

## 5. Discussion

The Iterative Shifting Disaggregation (ISD) algorithm is carried out in an iterative two-phase process; where first a prediction phase uses multiple linear regression to generate high-frequency time series based on the relationship between the sensor-sourced low-frequency series and high-frequency exogenous correlated variables. Then second, load profiles are used in a piecewise linear update phase to “shift” or redistribute the low-frequency consumption observations among the underlying high-frequency periods within each low-frequency measured interval. This two-step process repeats, with each subsequent prediction phase modeling the updated estimates produced in the preceding update phase.

The ISD algorithm relies on the use of highly correlated exogenous variables measured at the desired target frequency of $\hat{y}$ in the prediction phase. Without such variables, the multiple linear regression fails to generate accurate high-frequency estimates, leading to incorrect distribution of low-frequency consumption observations in the ISD shifting step. For temperature-sensitive datasets, exogenous variables such as HDD and CDD capture the seasonal and daily variations in gas consumption. These variables directly inform the load-shifting process by redistributing consumption across high-demand periods, such as cold days. For datasets with lower temperature sensitivity, alternative variables, such as weekly or monthly usage trends, may better capture underlying patterns. Incorporating flexible transformations of weather data can also enhance the algorithm’s adaptability to varying degrees of temperature dependence. It is the careful selection of these exogenous variables using domain knowledge that make the ISD algorithm perform well.

We used eight daily weather-based time series as our exogenous variables. All forecasting methods show a strong correlation with the actual values, indicating they are reasonable models. The eight exogenous variables in Table 3 correspond to: one constant (Var1), a linear trend (Var2), Heating Degree Days HDD65 and HDD55(Var3−4), Cooling Degree Day CDD65 (Var5), and four seasonal variables (Var6−9) used to capture yearly seasonality [2]. The same variables were used as inputs to all other disaggregation algorithms except for NAV.

The ISD algorithm does not have more information than TSR and GLS disaggregation methods yet distinguishes itself as the best disaggregation approach in the case studies presented in Section 4. This success is attributed to the ISD constrained shifting to iteratively redistribute low-frequency load observations among underlying high-frequency periods. The proportion of load shifted is dependent on the correlation between all independent exogenous variables and the underlying gas consumption series per-ISD-iteration. In the case studies in Section 4, which use extremely simple natural gas demand forecasting models, the effectiveness of distributed shifting is dependent on each dataset’s temperature sensitivity. The correlation between the temperature sensitive exogenous variables and the underlying gas consumption series per-ISD-iteration are shown in Table 3.

The Var1 constant variable (corresponding to baseload) is not included in the correlation analysis of Table 3. For the more temperature-sensitive Operating Area dataset, the strongest correlations are observed with temperature-related variables (Var3 and Var4), which consistently exceed 0.9 as the iterations progress. The Cooling Degree Day variable (Var5) shows a moderate negative correlation, while the seasonal variables (Var6−9) exhibit varying levels of positive correlation, indicating their influence on gas consumption patterns.

In contrast, the non-temperature-sensitive Residential 100 dataset shows weaker correlations overall, especially for Var2 and Var5, which reflect the lesser impact of temperature on gas consumption in this area. However, temperature-related variables still maintain moderate to strong correlations, indicating their relevance even in non-temperature-sensitive contexts as seen in Table 3. Since we are fitting with this model as opposed to forecasting, the expected insignificant t-statistics are not as important in this context. The seasonal variables again show variable influence, particularly in later iterations.

These results support the effectiveness of the ISD algorithm in refining the correlation between the exogenous variables and gas consumption over successive iterations, particularly in temperature-sensitive regions. This is especially evident when comparing ISD estimates against GLS and TSR algorithm output (Figure 12) and numerical results (Table 1). Figure 12 shows the GLS algorithm underestimates consumption during peak heating seasons, and that the TSR algorithm more accurately follows the actual values during these periods of high volatility. Table 1 further confirms this with 4.3% WMAPE improvement of TSR over GLS, as well as 1.4% WMAPE improvement of ISD over TSR disaggregation results. Both GLS and TSR minimize the same cost function, but TSR, tailored for natural gas disaggregation, performs better than the generic GLS implementation.

The TSR method operates over a single series at a time and is effective in disaggregating consumption in temperature-sensitive areas. However, the TSR algorithm fails to maintain the reconciliation constraint (7), and the sum of the intramonthly (disaggregated) days does not sum to the original monthly totals. This failure to meet the reconciliation constraint is intentionally violated by the authors so accuracy on days with extreme weather events may be more accurate [2]. This indicates how important accurate daily estimates are to the local distribution companies’ distribution systems in extreme winter months. This is again observed in Table 2, where we observe a smaller spread of RMSE metrics for each algorithm but significant differences in WMAPE, indicating more accurate results were calculated for the ISD winter months.

Our results show that the ISD algorithm similarly can extract exogenous variable volatility as TSR and accurately redistribute gas consumption while maintaining the coherence constraint. This redistribution of load is based on the inter- and intra-series interactions of each dependent variable and the rate of gas consumption. The stronger the correlation between the low-frequency observed gas intervals and high-frequency independent correlated variables, the more accurate ISD disaggregate estimates become.

Under certain conditions, the ISD disaggregation algorithm will not perform well. As seen in the Table 2 results, the disaggregating the Residential 100 dataset was too dynamic of a problem to iteratively redistribute the gas consumption of 100 customers simultaneously and more accurately than the NAV persistence model. The variability introduced by considering individual residential consumption patterns outweighed and smoothed the effects of the ISD constrained load shifting. Still, ISD is the second-most accurate algorithm disaggregating the Residential 100 dataset, despite the large number of time series having lower correlation with exogenous variables.

The ISD algorithm demonstrates linear computational complexity with respect to the number of time series N and the number of iterations I. The naïve disaggregation step (Step 1) scales with O(N), as does the prediction phase in Step 2, where multiple linear regression models are fit independently for each series. The update phase in Step 3 introduces additional complexity, O(I⋅N), due to iterative adjustments across intervals.

For large-scale applications, such as disaggregating thousands of time series, computational efficiency can be enhanced by parallelizing Steps 2 and 3. Distributed computing frameworks can further accelerate processing by assigning time series to independent computational nodes. Additionally, adaptive convergence criteria may reduce the number of iterations I required, balancing accuracy and runtime.

Empirical tests suggest that ISD can handle datasets of up to 10,000 time series efficiently on modern hardware. However, optimizing memory usage and minimizing data transfer overhead remain critical areas for improvement.

## 6. Conclusions

The Iterative Load Shifting (ISD) disaggregation algorithm is more accurate than the naïve, Generalized Least Squares (GLS), and Time Series Reconstruction (TSR) alternatives when evaluated on the operating area and individual residential gas consumption datasets. Major contributions of this work include the ISD algorithm’s ability to disaggregate multiple, nonuniformly spaced time series with overlapping intervals into a single daily representation. This is achieved while maintaining the coherency constraint posed in Section 2.

Future work involves refining the algorithm to better suit the natural gas distribution problem. For example, we can enhance the naïve disaggregation process in Step 1 by incorporating time series reconciliation techniques. This will yield more accurate forecasts while maintaining coherence. We hypothesize that leveraging the same factors driving gas consumption in the design matrix X can enhance a priori reconciliation. Other future work could explore ISD’s applicability beyond natural gas consumption. The algorithm’s core iterative redistribution framework may extend to other domains where irregularly sampled data must be transformed into high-frequency representations, such as water demand forecasting or integrating multi-source sensor data for environmental monitoring. However, specialized adaptations would be necessary for applications outside of time series disaggregation, such as image processing, to ensure coherence constraints align with domain-specific requirements.

The ISD algorithm has potential applications beyond natural gas consumption, including electricity usage, water demand, and traffic flow. For each domain, the algorithm can be adapted by selecting domain-specific exogenous variables. For electricity disaggregation, exogenous variables such as solar irradiance, weather patterns, and grid data could be incorporated. Similarly, water demand forecasting might use precipitation, temperature, and population metrics, while traffic flow modeling could integrate data on road capacity, vehicle counts, and urban development. By tailoring constraints and selecting domain-specific exogenous variables, the ISD algorithm can address a wide range of time series disaggregation challenges in diverse fields.

Iterative-update disaggregation algorithms such as ISD focus on maintaining temporal coherence, ensuring high-frequency estimates align with low-frequency totals. This makes ISD particularly effective for applications requiring strict consistency, such as energy distribution. In contrast, forecast reconciliation techniques optimize forecasts across hierarchical levels, potentially achieving higher accuracy by relaxing coherence constraints.

Each method has unique strengths: ISD excels in maintaining aggregation consistency, while reconciliation techniques offer flexibility in improving forecast precision across multiple levels. A hybrid approach could integrate ISD’s coherence-preserving structure with reconciliation’s ability to refine accuracy, especially for periods of high variability or complex hierarchies. This could improve forecast quality and computational efficiency in diverse applications.

Additionally, we plan to analyze the trade-offs between iterative-update disaggregation algorithms like ISD and optimal forecast reconciliation techniques. Our load-shifting algorithm introduces inter-series interaction by iteratively modeling exogenous variables with gas load. In contrast, reconciliation techniques can summarize these interactions optimally across a predefined hierarchy [27]. Combining these approaches can balance coherence and accuracy [28]. Incorporating reconciliation-based load-shifting in Step 3 could also improve the speed of the ISD disaggregation algorithm and potentially enhance forecast accuracy.

## Figures and Tables

**Table 1:** *RMSE*, *MAPE*, and *WMAPE*, and coherency indicator for NAV, TSR, GLS, and ISD disaggregation algorithms applied to Operating Area dataset.

Method	Coherent	*RMSE* [Dth]	*MAPE* [%]	*WMAPE* [%]
NAV	Yes	153.9	18.8	16.9
TSR	No	71.5	11.4	8.6
GLS	No	101.24	16.9	12.9
ISD	Yes	53.3	9.7	7.2

**Table 2:** *RMSE*, *MAPE*, *WMAPE*, and coherency indicator for NAV, TSR, GLS, and ISD disaggregation algorithms applied to Residential 100 dataset.

Method	Coherent	*RMSE* [Dth]	*MAPE* [%]	*WMAPE* [%]
NAV	Yes	20.32	37.14	33.82
TSR	No	11.62	18.45	13.73
GLS	No	15.89	23.96	19.43
ISD	Yes	6.61	11.13	9.07

**Table 3:** Correlation between independent exogenous variables and underlying gas consumption series.

Iteration No.	Exogenous Variable							
	Var2	Var3	Var4	Var5	Var6	Var7	Var8	Var9
	Temperature Sensitive LDC Operational Area (Operating Area)							
1	0.29	0.92	0.90	−0.53	0.61	0.09	0.21	0.14
2	0.25	0.93	0.92	−0.37	0.75	0.19	0.35	0.16
⋮	⋮	⋮	⋮	⋮	⋮	⋮	⋮	⋮
10	0.21	0.98	0.97	−0.44	0.68	0.17	0.41	0.37
	Non-Temperature Sensitive LDC Operational Area (Residential 100)							
1	0.03	0.72	0.75	−0.26	0.59	0.17	0.41	0.27
2	−0.06	0.76	0.61	−0.19	0.74	0.22	0.35	0.34
⋮	⋮	⋮	⋮	⋮	⋮	⋮	⋮	⋮
10	−0.11	0.81	0.83	−0.33	0.43	0.27	0.26	0.31

## Data Availability

The original contributions presented in the study are included in the article; further inquiries can be directed to the corresponding author.

## Appendix A

Each function introduced in the pseudocode corresponds to a key computational step in the ISD algorithm. The following descriptions clarify their purpose and reference relevant sections of the manuscript.

**Algorithm A1: Pseudocode for the Iterative Shifting Disaggregation (ISD) Algorithm**		
# **Inputs:** # A, B, C, …—Multiple low-frequency, inconsistently spaced time series for disaggregation. # X—P independent correlated variables measured at the target high frequency # n_LR_Models—Number of linear regression models trained # n_Disagg_Cycles—Number of disaggregation iterations # α—Weight controlling how error is redistributed # **Output:** # y_hat—Estimated high-frequency, consistently spaced disaggregated time series		

# **Step 1: Initial Naïve Disaggregation** a = naïve_disaggregate(A) # Evenly distribute low-frequency values of A b = naïve_disaggregate(B) # Evenly distribute low-frequency values of B c = naïve_disaggregate(C) # Repeat for additional sources y_hat = aggregate_series(a, b, c) # Initialize y_hat as the aggregate all naïvely disaggregated series on day d # **Step 2: Construct Design Matrix** X = construct_design_matrix(*X*) # Create $N_{D}\;x\;P$ matrix of transformed independent variables $x_{1},\;x_{2},\;\ldots \;x_{P}$		
# **Step 3: Iterative Disaggregation Process**		
for i in range(n_LR_Models): # Multiple regression models trained		
	# Prediction Phase: Estimate high-frequency values beta = train_regression_model(X, y_hat) # Fit regression model y_tilde = X @ beta # Compute predicted values	
	for j in range(n_Disagg_Cycles): # Iterative shifting process	
		for k in range(nIntervals): # Iterate over all intervals. # len(A) = len(B)= len(C) assumes all series on the same time domain
		# Update Phase: Adjust estimates within each interval y_hat_k = remove_interval(y_hat, k) # Temporarily remove kth interval from # y_hat. Remaining values in y_hat interval k represent all other aggregate # consumption (ex: consumption from B, C, …) interval_error = y_tilde − y_hat_k # Compute error signal (remaining consump- # tion w/kth interval removed) adjusted_values = compute_interval_adjustment(interval_error, y_hat_k)# Scale # adjustment to interval error y_tilde = apply_adjustments(y_tilde, adjusted_values) # Redistribute interval # estimate with weighting α. y_hat = restore_interval(y_hat, y_hat_k, k) # Reinstate updated interval to # the aggregate.
# **Final Output: Refined high-frequency series** return y_hat		
Appendix A Method Descriptions: **construct_design_matrix(X)** This function constructs the design matrix, which organizes exogenous variables used in the regression model. The matrix consists of rows representing daily observations and columns containing predictor variables such as HDD, CDD, and HDDW. Section 3.2 explains how this matrix is formed and why it is necessary for estimating high-frequency gas consumption patterns. **train_regression_model(X, y_hat)** This function trains a multiple linear regression model to estimate daily consumption using the exogenous variables stored in the design matrix. It calculates the relationship between the predictor variables and the initial disaggregated series, producing refined estimates. Section 3.2 describes this regression step, detailing how external factors influence gas usage. An implementable version of this function can be found in [29]. **remove_interval(y_hat, k)** This function temporarily removes interval k from the aggregated time series. By isolating the remaining data, the algorithm can analyze how much of the total high-frequency estimate is attributable to the removed interval. This step is necessary for the iterative adjustment process described in Section 3.3, which refines individual interval estimates while maintaining the overall structure of the data. **compute_interval_adjustment(interval_error, y_hat_k)** This function calculates how much each interval should be adjusted based on the error signal from the regression model. The adjustment is scaled to ensure that changes to one interval do not distort the overall time series structure. Section 3.3 explains how these corrections are applied iteratively to improve disaggregation accuracy. **apply_adjustments(y_tilde, adjusted_values)** This function redistributes interval-level corrections across the high-frequency predictions. Adjustments are weighted to ensure that each interval remains proportional to the original low-frequency observation while still incorporating the refined estimates from the regression model. Section 3.3 discusses how this step gradually improves the accuracy of the disaggregated series. **restore_interval(y_hat, y_hat_k, k)** This function restores the corrected interval back into the aggregated series after adjustments are made. Once an interval has been updated, it is reintegrated into the full dataset to maintain continuity across all sources. Section 3.3 explains how this ensures that ISD converges toward a stable high-frequency estimate. Each of these functions corresponds to a specific step in Algorithm 1 and is referenced throughout Section 3 of the manuscript. While the precise implementation may vary depending on the dataset and software environment, these descriptions outline the necessary components for applying ISD in practice.		

## References

[1] Askari S., Montazerin N., Zarandi M.H.F. (2016). High-Frequency Modeling of Natural Gas Networks from Low-Frequency Nodal Meter Readings Using Time-Series Disaggregation. IEEE Trans. Ind. Inform..

[2] Vitullo S.R., Corliss G.F., Adya M., Nourzad F., Brown R.H. (2013). An Algorithm for Disaggregating Temporal Natural Gas Consumption. Can. Appl. Math. Q..

[3] De Alba E. (1988). Disaggregation and Forecasting: Bayesian Analysis. J. Bus. Econ. Stat..

[4] Sax C., Steiner P. (2013). Temporal Disaggregation of Time Series. R J..

[5] Moauro F., Savio G. (2005). Temporal Disaggregation Using Multivariate Structural Time Series Models. Econom. J..

[6] Wickramasuriya S.L., Athanasopoulos G., Hyndman R.J. (2015). Forecasting Hierarchical and Grouped Time Series through Trace Minimization.

[7] Kaefer P.E. (2015). Transforming Analogous Time Series Data to Improve Natural Gas Demand Forecast Accuracy.

[8] Hong T. (2013). Energy Forecasting: Past, Present, and Future. Foresight Int. J. Appl. Forecast..

[9] Suganthi L., Samuel A.A. (2012). Energy Models for Demand Forecasting—A Review. Renew. Sustain. Energy Rev..

[10] Wei W., Stram D. (1990). Disaggregation of Time Series Models. Wiley R. Stat. Soc..

[11] Ayodeji S.D., Olagunju M.A. (2012). Temporal Disaggregation of Time Series Data: A Non-Parametric Approach. Math. Theory Model..

[12] Chan W.-S. (1993). Disaggregation of Annual Time-Series Data to Quarterly Figures: A Comparative Study. J. Forecast..

[13] Shumway R., Stoffer D. (2017). Time Series Analysis and Its Applications with R Examples.

[14] Chow G.C., Lin A. (1971). Best Linear Unbiased Interpolation, Distribution, and Extrapolation of Time Series by Related Series. Rev. Econ. Stat..

[15] Guerrero V.M., Nieto F.H. (1999). Temporal and Contemporaneous Disaggregation of Multiple Economic Time Series. Off. J. Span. Soc. Stat. Oper. Res..

[16] Tamba J.G., Essiane S.N., Sapnken E.F., Koffi F.D., Nsouandélé J.L., Soldo B., Njomo D. (2018). Forecasting Natural Gas: A Literature Survey. Int. J. Energy Econ. Policy.

[17] Wei L., Jin L. (2024). Collaborative Neural Solution for Time-Varying Nonconvex Optimization With Noise Rejection. IEEE Trans. Emerg. Top. Comput. Intell..

[18] Xiao L., Dai J., Lu R., Li S., Li J., Wang S. (2020). Design and Comprehensive Analysis of a Noise-Tolerant ZNN Model With Limited-Time Convergence for Time-Dependent Nonlinear Minimization. IEEE Trans. Neural Netw. Learn. Syst..

[19] Liao B., Han L., Cao X., Li S., Li J. (2024). Double Integral-Enhanced Zeroing Neural Network with Linear Noise Rejection for Time-Varying Matrix Inverse. CAAI Trans. Intell. Technol..

[20] Fakoor M., Corliss G.F., Brown R.H. Prior Day Effect in Forecasting Daily Natural Gas Flow from Monthly Data. Proceedings of the 2018 IEEE Power & Energy Society General Meeting (PESGM).

[21] Merkel G.D., Povinelli R.J., Brown R.H. (2018). Short-Term Load Forecasting of Natural Gas with Deep Neural Network Regression. Energies.

[22] Vitullo S.R., Brown R.H., Corliss G.F., Marx B.M. (2010). Mathematical Models for Natural Gas Forecasting. Can. Appl. Math. Q..

[23] Huntington H.G. (2007). Industrial Natural Gas Consumption in the United States: An Empirical Model for Evaluating Future Trends. Energy Econ..

[24] Timmer R.P., Lamb P.J. (2007). Relations between Temperature and Residential Natural Gas Consumption in the Central and Eastern United States. J. Appl. Meteorol. Climatol..

[25] Akaike H. (1969). Fitting Autoregressive Models for Prediction. Ann. Inst. Stat. Math..

[26] Vitullo S. (2011). Disaggregating Time Series Data for Energy Consumption by Aggregate and Individual Customer.

[27] Hyndman R.J., Ahmed R.A., Athanasopoulos G., Shang H.L. (2011). Optimal Combination Forecasts for Hierarchical Time Series. Comput. Stat. Data Anal..

[28] Quinn C.O., Corliss G.F., Povinelli R.J. (2024). Cross-Temporal Hierarchical Forecast Reconciliation of Natural Gas Demand. Energies.

[29] MATLAB Regression Operator. https://www.mathworks.com/help/stats/regression-and-anova.html.

